# Antibiotic Infographics Available on the Internet: Documentary Quality, Purpose, and Appropriateness as Educational Tools on Antimicrobial Resistance

**DOI:** 10.3390/antibiotics12030462

**Published:** 2023-02-24

**Authors:** Elsa López-Pintor, Aitana Gómez-Ramos, Javier Sanz-Valero

**Affiliations:** 1 Department of Engineering, Area of Pharmacy and Pharmaceutical Technology, Miguel Hernandez University, 03550 San Juan de Alicante, Spain; 2 CIBER of Epidemiology and Public Health, CIBERESP, 28029 Madrid, Spain; 3 Dissemination and Research and Services Area of the National School, Occupational Medicine of the Carlos III Health Institute, 28029 Madrid, Spain

**Keywords:** antibiotics, infographic, access to information, communication and education materials, copyright, antimicrobial resistance, WHO, Global Action Plan on AMR

## Abstract

Antimicrobial resistance is a major threat to global health in the 21st century. In the age of the internet and social media, infographics may constitute an effective educational resource for transmitting complete messages about antibiotics and antimicrobial resistance and driving behavioural change. We aimed to evaluate the infographics on antibiotics available on the internet in terms of their documentary quality, purpose, and appropriateness as educational tools for explaining the strategic lines defined in the World Health Organization Global Action Plan on Antimicrobial Resistance (GAP-AMR) and for conveying the One Health concept. We obtained the infographics for this cross-sectional study on 4 March 2021 by searching the terms “infographic” and “antibiotic” in Google Images. We verified infographic documentary quality by analysing the image, authorship, title, structure, date, and licence. To evaluate the purpose, we determined whether it coincided with one of the strategic objectives set out in the GAP-AMR. The degree of appropriateness depended on the type of key awareness message on antibiotic use. After obtaining these results, we performed a cross-sectional evaluation to determine how successfully these infographics conveyed the One Heath concept. We selected 247 infographics from 518 references. Of the included infographics, 97 (39%) were produced by public institutions; 58 (23%) read from left to right; 142 (57%) had an educational purpose; 156 (63%) focused on humans; 140 (57%) were subject to copyright; and 97 (39%) had no licence of any type. Almost one quarter (*n* = 57; 23%) included no key message on proper use of antibiotics. Infographics that included an author/promoter had a significantly higher mean number of messages that those without disclosure of authorship (1.67 vs. 0.50; *p* < 0.001). The infographics on antibiotics available on the internet are of moderate general quality. Most are produced by public institutions and have a clear and readable layout, but very few have a Creative Commons license to enable their reuse as informative material. The most common purpose is to improve awareness and understanding of antimicrobial resistance; few infographics focus on the remaining four strategic objectives of the GAP-AMR. It would be useful for authors of educational infographics on antibiotics to promote key messages related to antimicrobial resistance and the One Health concept, in accordance with the first objective of the WHO GAP-AMR.

## 1. Introduction

Antimicrobial resistance is a major threat to global health in the 21st century, as it compromises the therapeutic efficacy of antimicrobial drugs in combating the threats posed by infectious diseases in medical and veterinary practices worldwide [1]. Some 700,000 people are killed every year by drug-resistant infections [2]. International bodies such as the World Health Organization (WHO), the United Nations (UN), and the World Bank predict that antimicrobial resistance will continue to worsen unless we change how we develop and use antibiotics [3]. The consequences of resistance include prolonged disease duration, higher transmission of infections, increased morbidity and mortality, and substantial economic and social costs [4].

In 2015, the WHO approved the Global Action Plan on Antimicrobial Resistance (GAP-AMR), which sets out five strategic objectives for combating bacterial resistance [5]. These objectives have been adapted to national action plans in most countries [6]. To achieve them, experts are advocating a One Health approach that encompasses human, animal, and environmental health. This multisectoral approach requires all sectors involved to understand their role and assume responsibilities in a coordinated manner to cover all aspects affecting antimicrobial resistance.

The first of the five strategic objectives proposed by WHO is to “improve awareness and understanding of microbial resistance through effective communication, education and training” [5]. Therefore, the WHO considers that countries should focus on communication first and foremost in the fight against bacterial resistance. Unfortunately, awareness campaigns on antibiotics have been largely unsuccessful [7], perhaps as a result of hierarchical strategies, with information transmitted from experts to the general public [8], or perhaps because messages are not reaching their target audience [7]. More evidence is needed regarding the optimal method of spreading information about appropriate antibiotic use, but working in a less hierarchical manner could break down barriers to understanding complex and potentially emotive issues such as antibiotic awareness and stewardship [8] Bacterial resistance is a unique challenge for health communication experts, as their messages have to reach a heterogeneous target audience (doctors, pharmacists veterinarians, stockbreeders, and the general public) and change the behaviour of all actors involved [9]. Meeting this challenge requires new mitigation tactics and communication strategies [9]. The increased use of social media has transformed the paradigm of information sharing by providing innovative communication opportunities, as users can participate and create content rather than merely receiving information [10]. For example, the social networking service Twitter has been used as a learning tool in the field of medicine, as well as for monitoring diseases and managing outbreaks. Specifically, in the area of antibiotics, key opinion leaders such as institutions, medical journals, physicians and pharmacists can use Twitter to spread the AMR message [8,10]. Mackenzie and colleagues [8] evaluated the impact of World Antibiotic Awareness Week and European Antibiotic Awareness Day in 2018, finding a positive association between the impact of the messages, measured as retweets, and the presence of media content such as images and videos. This association supports the use of infographics for conveying information on antibiotics.

An infographic is a visual composition of text and images with the potential to convey messages in a concise and practical way without compromising their scientific value. Thus, infographics may constitute an effective educational resource [11] for transmitting complete messages [12] and driving behavioural change [13]. In an infographic, image and text are usually complementary; when the image fails to convey the message, the text fulfils this function, and vice versa. High-quality, well-designed infographics can arouse interest in unfamiliar topics and are associated with greater reader preference [14]. For this reason, in the area of health, infographics are becoming an important resource for explaining care procedures, diseases, or use of medicines [15], as well as for disseminating scientific research [16] and public health information [17]. Thus, they could help to increase understanding or awareness of antimicrobial resistance among the relevant sectors and the general public [18]. Indeed, public and private institutions often use infographics to disseminate information on this topic [19].

Despite the potential and growing use of infographics as an educational tool, no study has adequately explored their use in the dissemination of messages about antibiotics and antimicrobial resistance. The aim of this study was to evaluate the infographics on antibiotics that are available on the internet in terms of their quality, purpose, and appropriateness as educational tools for explaining the strategic lines laid down in the WHO GAP-AMR, in the context of One Health. This information could help authors of educational infographics on antibiotics to adapt their content to the WHO guidelines and contribute to the primary objective of improving awareness and understanding of microbial resistance through effective communication, education, and training.

## 2. Materials and Methods

### 2.1. Design

Cross-sectional content analysis.

### 2.2. Search Strategy

We performed a simple search on 4 March 2021, using the Google Images search engine and applying the search strategy [“infographic” + “antibiotic”]. No geographic filter was established. We used Google because it was the market leader in the period 2010–2022, used by 83% of internet users [20].

We used the browser Google Chrome, deleting the browsing history beforehand to eliminate results induced by previous searches.

### 2.3. Inclusion Criteria

We included graphic documents from around the world containing a combination of text and synthetic images that transmitted information about antibiotics.

### 2.4. Exclusion Criteria

- Infographics unrelated to antibiotics.
- Graphic documents other than infographics.
- Illegible infographics.
- Broken links to infographics.

### 2.5. Unit of Analysis

Each of the recovered infographics that met the inclusion criteria constituted a unit of analysis.

### 2.6. Data Extraction and Storage

To prevent changes in the results and maintain the links to the selected images, we saved the search results in PDF format. From this document we could access each infographic through its URL.

Two authors (AGR and JSV) independently evaluated the relevance of the graphic documents. For the selection process to be considered valid, between-observer agreement (Kappa index) had to be greater than 0.80 (good or very good agreement). Provided that this condition was met, any discrepancies were resolved by consulting a third author (ELP) and subsequently reaching a consensus among all authors.

Google translate was used in order to understand the nuances of infographics in various languages. In the case of further doubts, we consulted a native speaker with a background in science.

### 2.7. Study Variables

Infographics have considerable communicative potential because readers can form an opinion on their content in a few seconds [21]. We hypothesised that high-quality infographics with suitable content are useful for disseminating messages about antibiotics and driving behavioural change, thus contributing to the first strategic objective of the WHO GAP-AMR (to improve awareness and understanding of AMR through effective communication, education, and training). Therefore, we defined two types of variables:

(1) Variables related to the documentary quality of the infographic.

- Type of image: visual format of the information that appears in the database.
  ○ Infographic.
  ○ Photograph.
  ○ Template for creating infographics.
  ○ Graph/table.
  ○ Other images (video, advertisement, online post, GIF, tag cloud, etc.).
- Authorship: person or institution that is responsible for or that endorses the infographic.
  ○ Public institution.
  ○ Private institution.
  ○ Public/private institution.
  ○ Individual.
  ○ Not identified.
- Layout of the infographic: reading order.
  ○ Left to right.
  ○ Top to bottom.
  ○ Clockwise or anticlockwise.
  ○ Centred (image in the centre with text branching out).
  ○ Random or mixed (no reading order or a combination of any of the above).
- Defects.
  ○ Small font size.
  ○ No key message.
  ○ Colour combination challenging to colour blind people.
  ○ Complicated or unattractive.
- Title: how the title relates to the infographic.
  ○ Self-explanatory (summarises the content of the infographic).
  ○ Defines objectives (indicates the purpose of the infographic).
  ○ Lack of concurrence (with the content of the infographic).
- Date: presence of the year of creation within the infographic. If readers know when infographics are created, they know whether the information before them is valid. This circumstance influences infographic reliability because readers aim to consult only the most up-to-date information.
  ○ Yes.
  ○ No.
- Licence: existence of transfer of rights to use the infographic.
  ○ Yes.
  ○ Yes, external (mentioned on the website but not within the infographic).
  ○ No.
- Type of licence.
  ○ Copyright (without transfer of rights).
  ○ CC BY (credit must be given to the creator).
  ○ CC BY-SA (credit must be given to the creator; adaptations must be shared under the same terms).
  ○ CC BY-ND (credit must be given to the creator; no derivatives or adaptations of the work are permitted).
  ○ CC BY-NC (credit must be given to the creator; only noncommercial uses of the work are permitted).
  ○ CC BY-NC-SA (credit must be given to the creator; only noncommercial uses of the work are permitted; adaptations must be shared under the same terms).
  ○ CC BY-NC-ND (credit must be given to the creator; only noncommercial uses of the work are permitted; no derivatives or adaptation of the work are permitted).
  ○ None mentioned.

(2) Variables related to awareness raising and education on bacterial resistance and the One Health concept.

- Context: target audience of the infographic.
  ○ General public.
  ○ Health professionals.
  ○ Stockbreeders or animal owners.
  ○ Members of administrations.
- Appropriateness: parameters or set of priorities used to judge the value of the infographic. To categorise this variable, we considered the key messages on antibiotics generated by the European Centre for Disease Prevention and Control [22], an Agency of the European Union) for the general public.
  ○ Message 1: antibiotics always with prescription.
  ○ Message 2: consult a healthcare professional.
  ○ Message 3: responsible use of medicines.
  ○ Message 4: more information (message recommending readers to consult other information).
  ○ None (none of the above messages).
- Purpose: to classify this variable, the infographics were grouped according to the five strategic lines set out in the WHO GAP-AMR and adopted at the 68th World Health Assembly in 2015 [5].
  ○ Improving awareness and understanding of antimicrobial resistance.
  ○ Optimising the use of antimicrobial medicines.
  ○ Reducing the incidence of infection.
  ○ Surveillance and research.
  ○ New antibiotics or alternatives.

After the main analysis, we determined whether the infographics promoted a multisectoral approach to fighting bacterial resistance (One Health concept) or contained any related message.

### 2.8. Data Analysis

For the categorical variables, we calculated absolute frequencies and relative frequencies (%): authorship, layout of the infographics, date of the infographics, existence and type of license, context, and purpose. For the quantitative variables, we calculated the mean, standard deviation (SD), median, range, and interquartile range (IQR): presence of messages (alerts).

We analysed the association between categorical variables using the chi-square test (association between presence of defects or presence of a date or presence of a licence and presence of an author/promotor). For quantitative variables, to test the significance of the difference in means between independent samples, we used the Student *t* test, and for more than two groups, we used analysis of variance (ANOVA) with Tukey’s test (to test all the differences, two by two between means of treatments of an experience): presence and number of messages in relation to presence of an author and/or institutional affiliation and/or appropriateness.

The level of significance was α ≤ 0.05 or all hypothesis tests.

For all statistical analyses, we used SPSS version 27.0 for Windows. We checked the data using double tables and corrected any errors by consulting the original data.

## 3. Results

**Figure 1:**
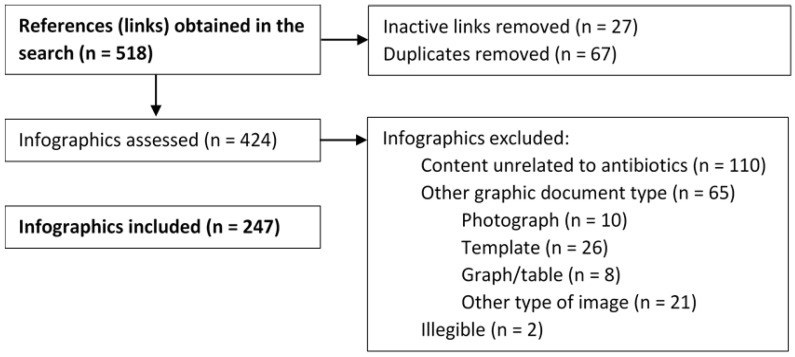
Infographic selection process.

The search recovered 518 references (links), of which 27 (5.21%) were inactive, meaning 491 graphic documents (94.79%) were accessible. After eliminating duplicate infographics, excluding infographics unrelated to antibiotics, and applying the inclusion criteria, we were left with 247 units of analysis (Figure 1). Between-observer agreement (Kappa statistic) regarding the relevance of the selected documents was 0.91 (*p* < 0.001). The noise (graphic documents other than infographics) in our results was not high compared with previous studies [3], but our search did recover many infographics unrelated to antibiotics. With the vast quantity of information available online, recovering all the relevant documents on a particular topic within certain thematic limits may prove impossible, either because they are highly dispersed or are not properly classified. This noise, an element that is not always controlled and that is rarely controllable, is an unavoidable part of the digital environment and is particularly problematic when trying to find information in the area of health sciences [23].

### 3.1. Authorship

Of the included infographics, 97 (39.17%) were produced by public institutions, which constituted the main type of author/promoter. Fourteen infographics (5.67%) were featured on personal websites, with no mention of the author/promoter within the infographic. In another 10 (4.05%), no author/promoter could be identified (Table 1).

In 147 infographics (59.51%), the affiliation was visible in the document itself. Table 2 presents the institutions responsible for five or more infographics.

### 3.2. Layout of the Infographics

The reading order of 122 (49.39%) of the infographics was left to right (Table 3).

The titles, which should serve the key function of attracting the target audience’s attention, were mostly self-explanatory (*n* = 153; 61.94%; Table 3). In contrast, 17 infographics (6.88%) had no title, and eight (3.24%) had a title that was unrelated to the content.

Some formal defects were found that made the infographics difficult to read and thus undermined their purpose (Table 3).

We found no association between the presence of an author/promoter and the presence of defects affecting readability (chi-square [χ^2^] = 2.51; degrees of freedom [df] = 2; *p* = 0.776).

### 3.3. Date of the Infographics

The year of creation was mentioned in 42 infographics (17.00%). Those created by public institutions were most likely to include this information (*n* = 24; 9.72%), though we found no association between the presence of a date and authorship (χ^2^ = 8.27; df = 4; *p* = 0.08).

Among the infographics that featured the year of creation, the most common year was 2016 (*n* = 10; 4.05%), and the earliest year was 2014 (*n* = 7; 2.83%).

### 3.4. Existence and Type of License

Of the infographics analysed, only 19 (7.69%) referenced a licence affecting the rights of use or distribution (within the document itself). Ten infographics (4.05%) had a Creative Commons licence (Table 4).

We found an association between the presence of an author/promoter and the mention of a licence (χ^2^ = 12.42; df = 2; *p* = 0.002), within the infographic (*n* = 18; 7.29%) or on the website (*n* = 131; 53.04%). However, we found no significant relationship between the presence of an author/promotor and the mention of a licence within the infographic alone (χ^2^ = 0.08; df = 1; *p* = 0.780).

### 3.5. Context

The included infographics mainly focused on the use of antibiotics in humans (*n* = 156; 63.16%). In addition, 36 (14.57%) were related to animals, and 55 (22.27%) were related to both humans and animals.

No infographics focused solely on the environment.

The target audience was distributed as follows: 184 (74.49%) infographics targeted the general public; 35 (14.17%) targeted health professionals; 26 (10.53%) targeted stockbreeders or animal owners; and two (0.81%) targeted members of administrative bodies.

### 3.6. Appropriateness

We analysed the presence of messages (alerts) aimed at helping the population to better understand the use of antibiotics (Table 5).

The mean number of messages per infographic was 1.62 (SD 0.08). The median was one message (IQR 2). The maximum number of messages was four, which applied to 24 infographics (9.72%), while the minimum was zero, observed in 57 infographics (23.08%).

We found a significant difference in the mean number of messages between infographics with and without an identified author/promoter (1.67 vs. 0.50; *p* < 0.001), between infographics produced by public institutions and those with no identified author/promoter (1.90 vs. 0.50; *p* = 0.006), and between infographics produced by public and private institutions (1.90 vs. 1.24; *p* = 0.002).

### 3.7. Purpose

The purposes of the infographics are grouped in Table 6 according to the five strategic lines set out in the WHO GAP-AMR.

The infographics centred on optimising antibiotic use had the highest mean number of messages, significantly more than the infographics focused on awareness and understanding of antimicrobial resistance (2.38 vs. 1.42; *p* < 0.001), on measures to reduce the incidence of infection (2.38 vs. 1.38; *p* = 0.02), on surveillance and research (2.38 vs. 0.80; *p* < 0.001), and on new antibiotics or alternatives (2.38 vs. 0.50; *p* = 0.002).

We found no association between type of institution and purpose of the infographics (χ^2^ = 19.34; df = 16; *p* = 0.2552), although infographics on improving awareness and understanding of antimicrobial resistance were mainly produced by public institutions (*n* = 53; 21.46%) and private institutions (*n* = 54; 21.86%).

### 3.8. Typical Infographic on Antibiotics

**Figure 2:**
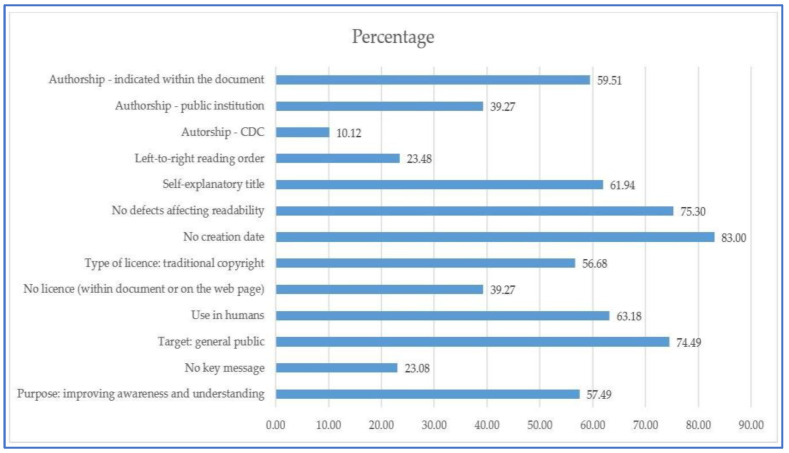
Summary of the most frequent characteristics of the included infographics. CDC: Center for Disease Control and Prevention.

With all the results, we summarised the most frequent characteristics of the infographics (Figure 2).

## 4. Discussion

Antimicrobial resistance is a serious public health problem worldwide. The WHO emphasises the importance of effective communication as a key strategy for tackling this problem [5]. Infographics are media content tools commonly used on the internet and social media to disseminate information on antibiotics or as part of international campaigns aimed at raising awareness of the proper use of antibiotics [24,25]; healthcare professionals also use them during consultations [24]. For this reason, appropriate, high-quality infographics could help to bring about the behavioural changes needed to reduce antibiotic resistance [25]. Ours is the first study to evaluate the documentary quality, purpose, and appropriateness of infographics available on the internet on antibiotics and microbial resistance. Our results demonstrate the usefulness of infographics in contributing to curbing global antimicrobial resistance, not only as a tool for disseminating antibiotic knowledge, but also as an interesting opportunity to enhance the dissemination of the One Health approach among the population. This observation is in line with the findings of Egan and colleagues [26], who showed that infographics are considered more trustworthy than documents with text alone and that their staged structure helps readers to remember messages. However, due to the rise in the use of social media, it is necessary to be more discerning about the visual material we produce, disseminate, and encounter [13].

We found most included infographics to be of moderate quality, mainly owing to deficiencies in their content. There was a general lack or scarcity of the ECDC key messages on the proper use of antibiotics and prevention of resistance [22,26]. Furthermore, we found that the infographics did not transmit the One Health multisectoral approach recommended to combat bacterial resistance [12], since they mostly focused on humans, and none focused on the environment. This is perhaps one of the main opportunities for improvement of infographics revealed by our findings: the need to promote messages related to the “One Health” concept in those infographics related to antibiotic use and the fight against bacterial resistance. The main purpose of the infographics included in our study was to educate the target audience. Therefore, our results can help guide authors of informative and educational infographics by making them aware of common mistakes to avoid.

Regarding the documentary quality of the infographics analysed in our study, most had an effective title, a logical layout, and no errors that could compromise readability. Therefore, they had the potential to offer precise information and a quick, easy, and enjoyable reading experience. This structural simplicity strengthens their power to ‘hook’ potential readers, and ensures the message is clear [27]. In line with Kiernan et al. [28], we found that a methodologically correct, well-structured infographic improves knowledge and trust.

The proportion of inactive links in our study was similar to that observed in other studies [29] and much lower than the proportion of shared resources lost after the first year of publication (11% according to one study [30]), which makes us suspect that antibiotic images tend to remain more active on the web compared to other resources.

Because very few of the analysed documents featured a creation date within the infographic itself, we could not determine the proportion of obsolete documents. Apart from this, the main opportunities for improvement in terms of documentary quality were related to correct licencing and authorship. The relationship between authorship and affiliation within documents found on the internet is an important indicator when analysing the quality of the information, although these factors are not always associated [29]. Since most of the infographics we studied were produced by public or private institutions, we were able to acknowledge and verify the validity of their content. Previous studies have proven that author identification and, in particular, the association of the author with a trusted institution are the most important quality criteria [30,31], as the internet is full of individual opinions that are often hidden behind non-existent, false, or anonymous figures.

One weakness of our study regarding authorship was that almost half of the included infographics mentioned no author or promoter within the document itself. This makes it difficult to reuse the infographic in another context, which is its very reason for being. Infographics with proper authorship are a valuable material for informing the corresponding target audience about antibiotics. The WHO recognises that infographics help to transmit health messages to the public through images and has a web page where this material can be downloaded for reuse [32], as does the US Centers for Disease Control and Prevention [33]. Given this circumstance, it is not surprising that these two institutions were responsible for most of the infographics included in our study. When we consider that health communication campaigns can influence the health behaviour of a population, that the public is very accustomed to the image culture, and that a range of technological supports are currently available, we can logically conclude that the sharing of infographics by health organisations is potentially one of the most effective strategies for achieving the dual objective of disseminating rigorous scientific content and educating the general public [15].

By studying the type of licence, we aimed to determine whether people who view infographics can reuse them in another context. We found this possibility was scarce or merely anecdotal, even in the case of infographics promoted by international institutions such as WHO. While this information often appears in the web pages hosting the documents, it ceases to be useful when the infographic is taken out of this space. As a result, the terms of reuse are difficult to access, as the Creative Commons licences allow authors to easily change the copyright terms and conditions from ‘all rights reserved’ to ‘some rights reserved’. These licences aim to provide an open-access valid legal framework [34]: a simple and standardised method for granting permission to the general public to share and use a creative work under the specified terms and conditions. This is relevant in the case of infographics because standard copyright terms and conditions prevent forms of reuse that most infographic authors not only allow but actively encourage, such as translation into other languages [35].

### Context, Purpose, and Appropriateness of Infographics on Antibiotics

The main target audience of infographics in our study was the general public, which is to be expected because the objective of this medium is to educate and simplify complex information, if possible in a striking and attractive format. As explained above, the visualisation of information through infographics is a key factor in the popularisation of technical and biomedical concepts. The public has an important role to play in the emergence, spread, and control of bacterial resistance to antibiotics [36], and awareness of this issue is crucial for reaching solutions. The use of infographics as an educational material in this field is logical, considering their capacity to transmit news, events, or dates in a visual format and thus stimulate readers’ interest and help them to understand complex or unfamiliar information [15]. For example, the COVID-19 pandemic has seen a rise in the use of infographics for the dissemination of information through social media, which is a prime example of the utility of this resource.

Although most infographics are aimed at the general public, they are also a useful resource for professionals, as our results show. Vilaplana-Camús found that researchers support the use of infographics, but that a lack of time and technical competence has resulted in low use of this resource [37]. Buljan and colleagues conducted a review to measure the usefulness of infographics in health communication (knowledge of health information presented, reading experience, and perceived ease of use), for professionals and non-professionals, comparing infographics with a scientific abstract and a plain language summary [38]. Although the authors found no difference in perceived knowledge resulting from the different formats, the participants (professionals and non-professionals) preferred the infographics, mainly owing to their readability.

Almost one in four infographics analysed in our study featured no key message related to the use of antibiotics (basic information to transmit to the population), the most important being the need for a medical prescription or consultation. Borgonha et al. [7] evaluated the impact of the 2018 UK television campaign ‘Keep Antibiotics Working’, which aimed to raise awareness of antibiotic resistance and reduce public demand for antibiotics. The authors concluded that the advert did not effectively communicate the seriousness of bacterial resistance and the importance of using antibiotics properly (e.g., it did not mention the need to use antibiotics only with a prescription) and thus did not motivate a change in the behaviour or attitude of most participants. Other previous studies on the health information available on Web 2.0 also found a lack of key messages [39,40]. While technology may provide more accessible means to creating elaborate infographics, it is essential to ensure critical messages remain coherent and enriching for the target audience.

Key messages that have reader approval and support help to ensure acceptance of and compliance with recommendations [41]. However, as we found, creators of infographics on antibiotics often miss this opportunity, undermining the power of infographics to educate the target audience. Although public institutions included these messages more frequently than other types of author/promoter, we believe they could have accomplished more.

Our results also showed that the inclusion of messages was more frequent in infographics directly related to the optimisation of antibiotic use, which may be because both the general public and professionals have greater awareness of certain recommended practices, such as using antibiotics only with a medical prescription and/or after a medical consultation or avoiding self-medication. These types of messages were probably included in the national programs on antimicrobial resistance implemented in various countries between 2017 and 2020 [41,42,43,44], primarily focused on rationalising the use of antibiotics in hospitals, in the community, and in farming and stockbreeding. In contrast, there were fewer key messages in infographics that aimed to educate the target audience about antimicrobial resistance and prevention of infection. One systematic review conducted by McCullough and colleagues in 2016 showed that the general public had incomplete knowledge and misperceptions about resistance to antibiotics and its causes [45]. It therefore seems necessary to promote the inclusion of key messages in infographics that focus on preventive measures and on lesser-known causes of increased resistance. In our opinion, the main messages on proper use of antibiotics should be clearly presented in all infographics that aim to educate readers or transmit information on antibiotics.

The infographics analysed in our study did not transmit the One Health multisectoral approach recommended to combat bacterial resistance [5], since they mostly focused on humans, and none focused on the environment. If infographics are to be used to educate and raise awareness among the population, they should explain the impact of the environmental sector on resistance to bacteria and on animal and human health. Including key messages specifically related to this concept could help to achieve the WHO objective of changing beliefs and making people aware of ways to improve this global health problem, beyond the rational use of antibiotics.

The main purpose of the infographics included in our study coincided with the first WHO strategic line, which is to be expected as infographics primarily serve to educate. Infographics are an excellent tool for approaching educational content and are particularly useful in health science teaching. Different fields of study, including health sciences, have used images to introduce and transmit knowledge [46]. However, educating and raising awareness is only the first of the five strategic objectives set out in the GAP-AMR [5]. Only 15% of the infographics dealt with any of the other strategic objectives, such as prevention of infections, epidemiological surveillance, or new antibiotics. If we assume that the infographics available reflect the level of implementation of the different strategic objectives, then the low prevalence of infographics on certain topics could highlight a need for greater dissemination of knowledge on aspects that are lesser known but equally relevant in the fight against antimicrobial resistance. The results of a study conducted by Alcíbar in 2018 suggest that information visualisation through infographics helped to popularise the technical and biomedical aspect of the latest Ebola epidemic, while also helping to construct a narrative of nature [47]. Sharing epidemiological results in an online resource favours the democratisation of data and, if conducted in a highly visual way, enables the various interested parties to participate and understand this information so that it can be used in decision-making processes [48].

A possible limitation of this study is the excess noise discussed previously. The low declaration of authorship observed in the infographics is another potential weakness. In addition, most infographics featured no year of creation to show readers whether the content was up to date. Another possible limitation is that we only evaluated the appropriateness of the infographics for the presence of messages aimed at educating the population to better understand antimicrobial use and resistance prevention. However, we did not evaluate the quality of the scientific content of each infographic. Although we have not detected it a priori, it could be the case that some infographics include inaccuracies or inappropriate content that do not contribute to reaching the objectives of the GAP-AMR. Further studies should therefore focus specifically on this.

## 5. Conclusions

In the age of social media, the effects of infographics for the dissemination of scientific information and public health education should be further studied to understand their full potential. In summary, the evaluation of the infographics on antibiotics recovered from the internet through Google Images showed that this format may constitute an effective educational resource for raising awareness of the global key objectives concerning antibiotic use. The quality of the documents recovered was considered moderate, mainly due to a lack of key messages about antibiotics and a failure to embrace the One Health approach. From a documentary point of view, key ingredients of an effective educational infographic on antibiotics are, apart from an eye-catching title, logical layout, and readability, a careful consideration of Creative Commons licencing and authorship information.

To improve appropriateness, bring infographics into line with the WHO GAP-AMR [12] 3, and promote effective communication on antibiotics and bacterial resistance, we propose the following improvements:

- Promote the One Health approach by including key messages on this concept.
- Promote the inclusion of information on the impact of the environmental sector on bacterial resistance.
- Promote infographics focused on other WHO strategic objectives, such as prevention of infections, epidemiological surveillance, and new alternatives to antibiotic treatment.

We also consider that increasing the use of Creative Commons licences would strengthen the informative potential of infographics.

Future research should test the impact of appropriate antibiotic infographics to improve awareness of antibiotics and bacterial resistance.

## Figures and Tables

**Table 1:** Authorship characteristics of infographics on antibiotics included in the study.

Authorship	*n* (%)
Public institution	97 (39.27%)
Private institution	96 (38.87%)
Public/private collaboration	30 (12.15%)
Personal (without endorsing institution)	14 (5.67%)
None mentioned	10 (4.05%)

**Table 2:** Institutions endorsing 5 or more infographics on antibiotics and visibility of the affiliation.

Institution	Mentioned in Infographic, *n* (%)	Mentioned on Web Page Only, *n* (%)	Total, *n* (%)
CDC	18 (7.29%)	7 (2.83%)	25 (10.12%)
WHO	14 (5.67%)	—	14 (5.67%)
ECDC	8 (3.24%)	3 (1.21%)	11 (4.45%)
PHAC	7 (2.83%)	—	7 (2.83%)
PEW	6 (2.43%)	1 (0.40%)	7 (2.83%)
Australian Govt	6 (2.43%)	—	6 (2.43%)
BioMérieux	5 (2.02%)	4 (1.62%)	1 (0.40%)

CDC: Centers for Disease Control and Prevention; WHO: World Health Organization; ECDC: European Centre for Disease Prevention and Control; PHAC: Public Health Agency of Canada; PEW: The Pew Charitable Trusts.

**Table 3:** Characteristics of the layout of included infographics: reading order, title and defects affecting readability.

Parameter	*n* (%)
Reading order	
Left to right	58 (23.48%)
Top to bottom	52 (21.05%)
Clockwise or anticlockwise	12 (4.86%)
Central	11 (4.45%)
Random/mixed	114 (46.15%)
Title	
Self-explanatory	153 (61.94%)
Defines objectives	69 (27.94%)
No title	17 (6.88%)
Not consistent with content	8 (3.24%)
Defects affecting readability	
No defects	186 (75.30%)
Small font size	16 (6.48%)
Complicated/unattractive	16 (6.48%)
No key message	15 (6.07%)
Inappropriate colours for colour blind readers	14 (5.67%)

**Table 4:** Mention of licence and types of copyright licences in the included infographics.

Parameter	*n* (%)
Mention of licence	
Within the infographic	19 (7.69%)
On the web page (not in the infographic)	131 (53.04%)
No information	97 (39.27%)
Type of licence	
Traditional copyright	140 (56.68%)
CC BY	4 (1.62%)
CC BY-NC-ND	3 (1.21%)
CC BY-NC-SA	2 (0.81%)
CC BY-NC	1 (0.40%)
No information	97 (39.27%)

CC: Creative commons licence; BY: credit must be given to the creator; NC: only non-commercial uses of the work are permitted; ND: no derivatives or adaptations of the work are permitted; SA: adaptations must be shared under the same terms.

**Table 5:** Analysis of the appropriateness of the included infographics according to the presence of key messages aimed at helping the population to better understand the use of antibiotics.

Type of Message	*n* (%)
Message 1	5 (2.02%)
Message 2	1 (0.14%)
Message 3	30 (12.15%)
Message 4	34 (13.77%)
Messages 1 + 3	17 (6.88%)
Messages 3 + 4	17 (6.88%)
Messages 1 + 4	15 (6.07%)
Messages 2 + 3	4 (1.62%)
Messages 1 + 3 + 4	22 (8.91%)
Messages 1 + 2 + 3	11 (4.45%)
Messages 2 + 3 + 4	9 (3.64%)
Messages 1 + 2 + 4	1 (0.14%)
Messages 1 + 2 + 3 + 4	24 (9.72%)
No message	57 (23.08%)

Message 1: antibiotics always with prescription; Message 2: consult a healthcare professional; Message 3: responsible use of medicines; Message 4: more information (message recommending readers to consult other information).

**Table 6:** Purpose of included infographics according to the five strategic lines set out in the World Health Organization Global Action Plan on Antimicrobial Resistance.

Strategic Line	*n* (%)
Improving awareness and understanding of antimicrobial resistance	142 (57.49%)
Optimising the use of antimicrobial medicines	68 (27.53%)
Reducing the incidence of infection	16 (6.48%)
Surveillance and research	15 (6.07%)
New antibiotics or alternatives	6 (2.43%)

## Data Availability

The full dataset and statistical code are available from the corresponding author.
